# Effect of Brief Guided Imagery on Short-Term Outcomes in Patients Undergoing First Elective Total Knee Arthroplasty: Randomized Controlled Trial

**DOI:** 10.3390/ijerph23030340

**Published:** 2026-03-08

**Authors:** Anat Kaplun, Omri Lubovsky, Ilia Prosso, Amit Sagi, Leonid Kalichman

**Affiliations:** 1 Department of Physical Therapy, Recanati School for Community Health Professions, Faculty of Health Sciences, Ben-Gurion University of the Negev, P.O.B. 653, Beer Sheva 84105, Israel; anatkaplun@gmail.com; 2 Department of Orthopedic Surgery, Barzilai Medical Center, Ashkelon 7830604, Israel; omril@bmc.gov.il (O.L.); iliaprosso@gmail.com (I.P.); dramitsagi@gmail.com (A.S.)

**Keywords:** postoperative recovery, brief guided imagery, postoperative pain, imagery-based therapy, non-pharmacological pain management, body–mind intervention

## Abstract

**Highlights:**

**Public health relevance—How does this work relate to a public health issue?**

- Brief guided imagery (GI) offers a low-cost, scalable, non-pharmacological strategy to address postoperative pain and functional limitations—major public health concerns among the growing population undergoing total knee arthroplasty (TKA).
- Improving early recovery after TKA can reduce healthcare utilization, enhance independence in older adults, and support better long-term quality of life.

**Public health significance—Why is this work of significance to public health?**

- This study demonstrates that a simple 2-min daily GI practice can meaningfully reduce postoperative pain and improve functional capacity, outcomes that directly influence rehabilitation success and patient well-being.
- The intervention’s accessibility and minimal resource requirements make it particularly valuable for health systems seeking effective adjuncts to standard postoperative care.

**Public health implications—What are the key implications or messages for practitioners, policy makers and/or researchers in public health?**

- Practitioners and rehabilitation teams can integrate brief GI into routine perioperative education to enhance early recovery without adding clinical burden.
- Policymakers and researchers may consider brief GI as a feasible component of multimodal pain management strategies and explore its broader implementation across surgical and chronic pain populations.

**Abstract:**

*Background*: Knee osteoarthritis, which is prevalent among older adults, often necessitates total knee arthroplasty (TKA) to alleviate pain and improve function. Postoperative pain and functional limitations remain significant challenges. Brief guided imagery (GI), a non-pharmacological intervention, shows promise in pain management but is underexplored in TKA patients. *Aim*: The aim of this study is to evaluate the effect of brief GI on postoperative pain, functional outcomes, and anxiety in patients undergoing their first elective TKA. *Methods*: Randomized controlled trial: 52 patients scheduled for first elective TKA were randomized to an intervention (brief GI plus standard care, n = 19) or control (standard care only, n = 23) group. Brief GI consisted of daily 2-min audio-guided exercises for up to 6 weeks after the operation. Outcome measures included pain intensity (NPRS), functional capacity (NFRS; WOMAC), and state anxiety (STAI). Assessments were conducted preoperatively (baseline), on the first postoperative day, weekly during the first five postoperative weeks, and again at the routine 5–6-week postoperative follow-up visit. *Results*: Of 52 enrolled participants, 42 completed the study. The intervention group reported significantly lower pain levels (NPRS) at weeks 2 (mean difference: 1.26, *p* = 0.042) and 5 (mean difference: 1.86, *p* = 0.004) compared to the control group, with a moderate effect size (Cohen’s d = 0.69–1.02). Functional outcomes (NFRS) were significantly better in the intervention group from week 1 through week 6 (*p* < 0.01). No significant differences were observed in WOMAC scores or STAI anxiety levels between groups. *Conclusions*: Brief GI, when integrated into postoperative care for TKA patients, significantly reduces pain and enhances functional outcomes over 6 weeks, though it does not affect anxiety levels. These findings support brief GI as a feasible adjunctive intervention for TKA recovery.

## 1. Introduction

Knee osteoarthritis is among the most common problems in older adults, with a global prevalence trend of site-specific osteoarthritis from 1990 to 2019 [1]. Total Knee Arthroplasty (TKA) is a common surgical procedure performed in patients with end-stage knee osteoarthritis who have failed conservative treatment [2] to improve function and ultimately reduce pain. However, some patients remain in pain and have functional problems after the operation [3,4]. Improving functional capacity and reducing postoperative pain in these patients are essential to their quality of life [5].

Guided imagery (GI) refers to a wide variety of techniques, including simple visualization of images and a series of verbal suggestions utilizing imagery, metaphors, storytelling, fantasy exploration, game playing, dream interpretation, drawing, and active imagination, where elements of the unconscious are encouraged to emerge as images [6].

GI is considered a practical, low-cost, and easily implemented therapeutic approach [7]. A recent comprehensive narrative review concluded that GI may contribute to reductions in pain intensity as well as psychological distress, including stress and anxiety [8]. The intervention is straightforward to administer in clinical settings and can be delivered using structured verbal instructions. A brief form of GI limits the exercise duration to approximately two minutes, making it feasible for routine daily use in both clinical and self-management contexts [8,9].

Kaplun et al. [10] conducted an exploratory controlled study to examine the impact of brief GI in 37 individuals diagnosed with fibromyalgia. Participants assigned to the intervention group (n = 18) practiced Colette’s brief GI protocol [9], whereas participants in the control group (n = 19) continued standard care. Between-group comparisons demonstrated significantly greater improvements in the intervention group across multiple domains, including pain severity, global activity level, mood, walking capacity, sleep quality, and overall enjoyment of life. Within-group analyses further revealed significant reductions in worst, least, and average pain scores among participants practicing brief GI, accompanied by improvements in daily functioning, mood, sleep, routine occupational tasks, ambulation, life satisfaction, and interpersonal relationships. Additional research conducted in a rheumatology outpatient population evaluated brief GI among patients with chronic pain conditions. Findings suggested that this intervention may reduce back pain intensity, alleviate anxiety, and enhance daily functioning in women with chronic low back pain [11]. Although preliminary, these data support the potential role of brief GI as an adjunctive non-pharmacological strategy for the management of chronic musculoskeletal pain. Importantly, these studies represent the limited number of high-quality investigations of brief GI in chronic pain populations, highlighting that the current evidence base is limited and that further high-quality research is needed to establish its effectiveness more conclusively.

There is also limited information on the effect of GI on postoperative pain. A previous study on GI in patients after TKA found significantly higher gait velocity (6 months after the operation). It significantly reduced the inflammatory and proliferative capacity of monocytes and cells, hair cortisol concentration, and the Western Ontario and McMaster Universities Arthritis Index (WOMAC) in the GI group, compared to the control group [12]. According to an integrative review by dos Santos [13], evidence suggests that GI combined with relaxation therapy can be a practical complementary approach to drug analgesia in managing postoperative pain [13].

Brief GI, lasting no more than 2 min, offers a practical and scalable intervention for postoperative care, yet its impact on TKA outcomes remains unexplored. The objective of this randomized controlled trial is to assess the influence of brief GI on postoperative anxiety, pain levels, and functional outcomes in patients undergoing their first elective TKA.

## 2. Methods

*Design*: Randomized controlled trial.

*Sample*: Patients scheduled for the first elective TKA procedure at the Barzilai University Medical Center, Ashkelon, Israel.

*Inclusion criteria:*

- Indication for primary elective TKA.
- Age 50–85 years, reflecting the typical demographic of patients undergoing elective knee arthroplasty, and ensuring that all outcome measures are validated for this age range.
- Functional independence prior to surgery, assessed through the orthopedic surgeon’s clinical evaluation and patient self-report, confirming independent ambulation (with or without a cane) and ability to perform basic activities of daily living.
- Ability to communicate in Hebrew, as all questionnaires and follow-up assessments were administered in their validated Hebrew versions.
- Possession of a smartphone with audio-playback capability, as the GI recordings were delivered digitally and no budget was available to provide alternative devices; all eligible patients approached during recruitment owned a smartphone.

*Exclusion criteria*: history of joint replacement, history of cerebrovascular accident, neurological or cognitive disorders.

*Sample size estimation*: We used “Power and sample size calculations” (version 3.0.12) software for sample size estimation. The main measurement for this trial was the numerical pain rating scale (NPRS), with a minimal detectable change of 2 [14]. The result showed that a sample size of 10 subjects in the intervention and 10 in the control group will be sufficient to reject the null hypothesis that the population means of the experimental and control groups are equal, with a probability (power) of 0.8. The Type I error probability associated with this test (NPRS) of this null hypothesis is 0.05. In this study, we used additional questionnaires, such as the WOMAC and the State-Trait Anxiety Inventory (STAI), and therefore aimed to recruit 40 patients: 20 for the intervention and 20 for the control. Sample size estimation for the primary outcome (NPRS) was performed in accordance with standard clinical trial methodology. Secondary outcomes (WOMAC and STAI) were included for exploratory purposes and were not individually powered.

*Ethical considerations*: Participation in the study was voluntary. All patients signed an informed consent form before participation. The study was approved by the Helsinki Committee of Barzilai Medical Center (0023-20BRZ).

*Recruitment*: About 2–3 weeks before surgery, patients were invited to meet with the anesthesiologist, orthopedic doctor, and nurse to plan the surgical procedure. The meetings were scheduled every week on the same day (Tuesdays). The orthopedic surgeon recruited patients for research. If the patients met the inclusion criteria, they received an explanation of the process and signed the consent form. Then, patients received preoperative education about functional outcomes and physical rehabilitation after TKA.

Patients were randomized to the intervention or control group using a computer-generated random sequence created by a researcher who was not involved in recruitment or data collection. Allocation concealment was ensured through the use of sequentially numbered, opaque, sealed envelopes. After obtaining informed consent and completing baseline assessments, the research assistant opened the next envelope in sequence to assign the participant to a group. This procedure minimized selection bias and ensured that group allocation remained concealed until the point of assignment.

*Intervention*: The surgical technique used in this study was kinematic TKA, an alignment approach that aims to restore the patient’s native knee kinematics by positioning components along the patient’s anatomical axes rather than a standardized mechanical alignment. A physical therapist arrives every week to provide preoperative education about the functional outcomes and physical rehabilitation after the surgery. Subjects in both groups received preoperative education. The education session was based on prior studies regarding patients’ expectations and educational needs [15,16,17,18].

In addition, subjects in the experimental group received detailed instructions on the brief GI technique. Instructions were performed in face-to-face sessions. Participants learned and practiced three exercises of the brief GI (“the breathing”, “the tree”, and “the olive oil” exercises). The breathing exercise involved visualizing calm inhalation and exhalation; the tree exercise focused on grounding imagery, and the olive oil exercise used sensory metaphors for relaxation. They received an audio recording of the exercises on their cell phones and were asked to practice brief GI exercises before and after surgery, regularly and daily, at least once a day. Adherence was encouraged and informally monitored through weekly telephone contacts, during which the researcher reminded participants to continue daily practice and confirmed ongoing use of the audio recording.

*Data collection*: T1: on the recruitment day and T2: the day after surgery. After the surgery, a researcher asked subjects by phone to rate their average pain in the last 24 h on an NPRS of 0–10 (0 = no pain, 10 = unbearable pain) once a week for 5 weeks (T3–T7). Participants also graded their function on a numerical function rating scale (NFRS) scale from 0–10, with 10 indicating they need help with everything and 0 indicating they don’t need help. In addition, the researcher reminded participants in the intervention group to use the GI exercises.

**Figure 1:**
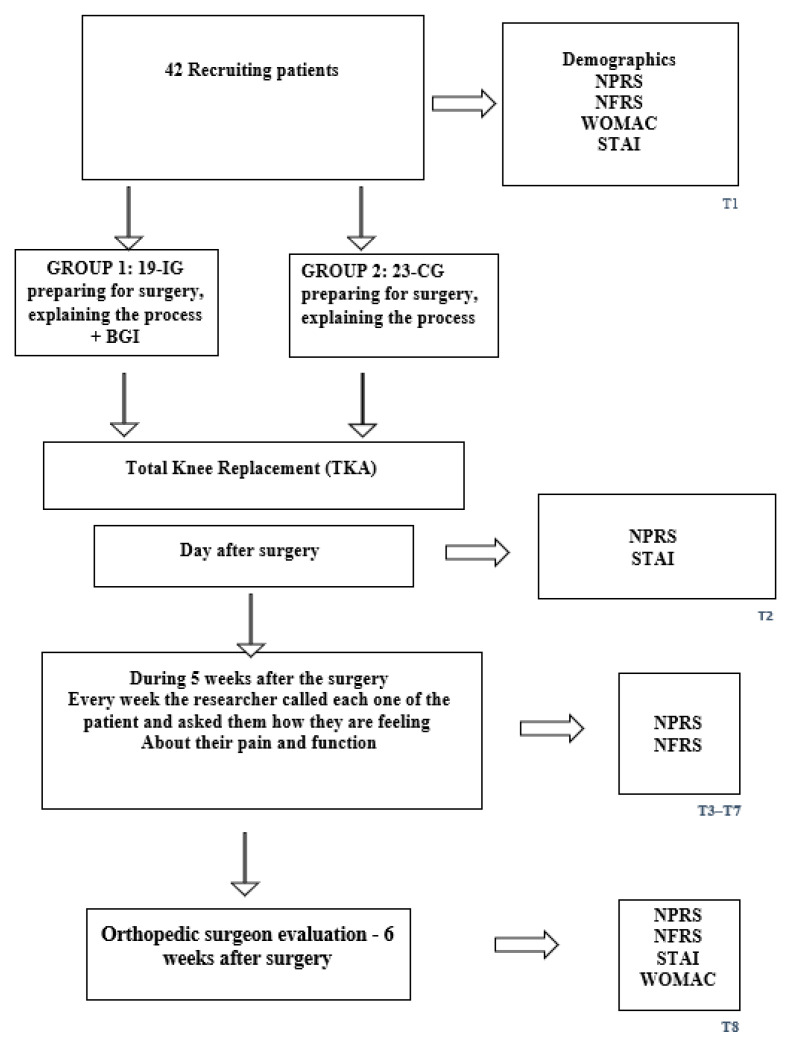
Flowchart of the study.

T8 on the day of the orthopedic surgeon’s postoperative evaluation, about 5–6 weeks after surgery (Figure 1).

*Outcome measures*: Subjects in both groups filled out the following questionnaires:

1. Pain intensity was assessed using the NPRS. A simple scale from 0 to 10 to measure pain intensity (average pain in the last 24 h). It is a reliable test, with an intraclass correlation coefficient of 0.85, and has high construct validity, with correlations with other pain measurements ranging from 0.88 to 0.93 [19,20].
2. Function was measured by NFRS, a simple scale from 0 to 10 to measure general function [21].
3. The Western Ontario and McMaster Universities Osteoarthritis Index (WOMAC) is one of the most extensively utilized patient-reported outcome measures for assessing symptom severity and functional impairment in individuals with hip and knee osteoarthritis. The instrument is self-administered and comprises 24 items organized into three domains. The pain subscale includes five questions addressing discomfort during ambulation, stair use, lying in bed, sitting or lying, and standing. The stiffness subscale contains two items that evaluate joint stiffness upon awakening and later in the day. The physical function subscale consists of 17 items assessing difficulty with activities of daily living, including stair negotiation, transitioning from sitting to standing, prolonged standing, bending, walking, transferring in and out of a car or bathtub, shopping, dressing (e.g., putting on or removing socks), rising from bed, toilet transfers, and performing light and heavy household tasks. The WOMAC has been translated into more than 65 languages and has undergone extensive cross-cultural validation.

The Hebrew adaptation of the WOMAC was evaluated in a cohort of 114 patients diagnosed with knee osteoarthritis. Test–retest reliability demonstrated moderate to strong stability, with Pearson correlation coefficients for individual items ranging from 0.55 to 0.78 (*p* < 0.01). Internal consistency was excellent, with Cronbach’s alpha coefficients of 0.97 at baseline assessment and 0.98 at follow-up. Construct validity was supported by statistically significant correlations (*p* < 0.01) between WOMAC scores and visual analog scale (VAS) ratings of pain and functional limitation. These findings indicate that the Hebrew version of the WOMAC is a reliable and valid instrument for assessing symptom severity and functional status among Israeli patients with knee osteoarthritis [22].

4. State anxiety was measured with the State-Trait Anxiety Inventory (STAI), considered a gold standard in measuring temporary anxiety in a specific situation. It consists of 20 statements expressing a range of emotional states, and responders are asked to rank them on a scale of 1 (not at all) to 4 (very much). The questionnaire demonstrated high reliability (0.86–0.95 across populations) and good validity, as evidenced by high correlations with other anxiety questionnaires (0.73–0.85) [23,24]. In its Hebrew version, Cronbach alpha was 0.91 [25]. State anxiety was evaluated preoperatively on the second day after surgery.

*Data Collection Schedule*: The measures taken in T1 include a demographic questionnaire, NPRS pain intensity (0–10; 0 = no pain, 10 = unbearable pain), NFRS (0–10; 0 = no need for help, 10 = need help at all functions), WOMAC, and STAI. The measures taken in T2 were NPRS and STAI. The measures taken in T3–T7 are NPRS and NFRS. The measures taken in T8 were NPRS, NFRS, STAI, and WOMAC (Figure 1). To minimize participant burden and accommodate postoperative clinical constraints, outcome measures were collected at different time points according to feasibility. All outcomes (NPRS, NFRS/WOMAC, and STAI) were assessed at baseline (T1) and at the 5–6-week postoperative follow-up (T8) when a complete in-person evaluation was possible. On the first postoperative day (T2), only NPRS and STAI were collected, as patients were in the immediate recovery phase and unable to complete longer functional questionnaires. During T3–T7, data were collected by telephone, allowing administration of NPRS and NFRS but not the longer WOMAC or STAI instruments. This schedule ensured consistent monitoring of pain and function while respecting clinical limitations and patient comfort.

*Data Analysis*: All statistical analyses were performed using SPSS (Version 29), with the significance level set at *p* < 0.05. Descriptive statistics were first used to summarize demographic and baseline clinical characteristics of the sample.

Normality of the main outcome variables (NPRS, NFRS, WOMAC, STAI) and demographic characteristics was assessed using the Shapiro–Wilk test.

All outcome variables demonstrated distributions appropriate for parametric analysis; therefore, repeated-measures mixed ANOVA was used to examine (1) the main effect of time and (2) the group × time interaction for each measure. When sphericity assumptions were violated, corrections were applied: Greenhouse–Geisser for NPRS and Huynh–Feldt for NFRS, as reflected in the reported degrees of freedom and *p*-values. No non-parametric tests were required.

Between-group differences at individual time points were evaluated using Bonferroni-adjusted post hoc comparisons to control for Type I error inflation. Effect sizes were reported as partial η^2^ for ANOVA models and Cohen’s d for pairwise comparisons to facilitate interpretation of the magnitude of observed effects.

## 3. Results

### 3.1. Participant Characteristics

Postoperative variables (e.g., complications and length of stay) are reported to describe the clinical course of the enrolled sample and were not part of the preoperative inclusion or exclusion criteria. Of 52 patients initially enrolled, 42 completed the study (19 in the intervention group and 23 in the control group). Ten participants dropped out due to reasons including cancellation of surgery (n = 5), postsurgical complications (patellar fracture, n = 1; neurological complication requiring additional surgery, n = 1), incompatible knee surgery (n = 1), voluntary withdrawal (n = 1), and technical issues (n = 1). Baseline characteristics (Table 1) showed no significant differences between groups in age (intervention: 69.9 ± 7.1 years; control: 72.4 ± 7.2 years; *p* = 0.27), education, marital status, number of children, country of origin, years of pain (intervention: 6.7 ± 8.9; control: 6.3 ± 4.3; *p* = 0.85), pain intensity (NPRS; intervention: 7.5 ± 2.0; control: 7.7 ± 2.2; *p* = 0.74), function (NFRS; intervention: 4.72 ± 3.01; control: 3.61 ± 3.01; *p* = 0.27), or physical activity (*p* = 0.083). Only demographic and clinical variables relevant to the interpretation of postoperative recovery were retained in Table 1 to maintain focus and avoid unnecessary personal or nationality-related information. However, a significant gender imbalance was observed, with fewer men in the intervention group (26.3%) compared to the control group (56.5%; χ^2^ = 3.867, *p* = 0.049). A post hoc ANCOVA adjusting for gender showed no significant interaction with pain or function outcomes (*p* > 0.05).

### 3.2. Pain Outcomes (NPRS)

The intervention group, receiving brief GI plus standard care, reported significantly lower pain levels (NPRS) compared to the control group (standard care only) from the second postoperative week onward (Table 2). Repeated-measures ANOVA revealed a significant group-by-time interaction (F (5.1, 183.65) = 2.36, *p* = 0.041, partial η^2^ = 0.06). Post hoc comparisons (Bonferroni-corrected) showed significant mean differences at week 2 (T4: 1.26, 95% CI [0.05, 2.48], *p* = 0.042, Cohen’s d = 0.69) and week 5 (T7: 1.86, 95% CI [0.63, 3.08], *p* = 0.004, Cohen’s d = 1.02), indicating moderate to large effects (Table 3). No significant differences were observed preoperatively (T1: *p* = 0.733) or in the first postoperative week (T2: *p* = 0.319, T3: *p* = 0.541). The overall time effect was significant (F (5.1, 183.65) = 27.22, *p* < 0.001, partial η^2^ = 0.43), reflecting pain reduction in both groups over 6 weeks.

### 3.3. Functional Outcomes (NFRS and WOMAC)

Functional capacity, which was assessed by the Numerical Function Rating Scale (NFRS), was significantly better in the intervention group compared to the control group from week 1 post-surgery (T3) through week 6 (T8; Table 2). A significant group-by-time interaction was observed (F (2.38, 92.84) = 6.29, *p* = 0.002, partial η^2^ = 0.14). Post hoc tests (Bonferroni-corrected) showed significant mean differences at all postoperative time points, with the largest at week 4 (T4: −2.691, 95% CI [−4.033, −1.348], *p* < 0.001, Cohen’s d = 1.22) and week 8 (T8: −2.483, 95% CI [−3.746, −1.220], *p* < 0.001, Cohen’s d = 1.15; Table 4). The overall time effect was significant (F (2.38, 92.84) = 21.91, *p* < 0.001, partial η^2^ = 0.36), indicating functional improvement in both groups.

In contrast, no significant differences were found in Western Ontario and McMaster Universities Arthritis Index (WOMAC) scores between groups (F (1, 37) = 0.76, *p* = 0.388, partial η^2^ = 0.02). A trend toward improvement was noted in the intervention group (T3: 0.39 ± 0.15 vs. control: 0.48 ± 0.17), but this did not reach statistical significance (*p* = 0.218).

### 3.4. Anxiety Outcomes (STAI)

State anxiety, as measured by the State-Trait Anxiety Inventory (STAI), showed no significant differences between groups preoperatively (T1: intervention: 20.00 ± 13.52; control: 16.65 ± 13.69; *p* = 0.98) or postoperatively (T2: intervention: 24.27 ± 12.08; control: 25.35 ± 11.59; *p* > 0.05; Table 2). Repeated-measures ANOVA indicated no group-by-time interaction (F (2, 66) = 0.78, *p* = 0.463, partial η^2^ = 0.02), though a significant time effect was observed (F (2, 66) = 5.79, *p* = 0.005, partial η^2^ = 0.15), reflecting a general reduction in anxiety post-surgery.

## 4. Discussion

### 4.1. Summary of Findings

This randomized controlled trial evaluated the impact of brief GI, a 2-min daily visualization intervention, on postoperative outcomes in patients undergoing their first elective TKA. The intervention group, receiving brief GI alongside standard care, demonstrated significantly lower pain levels, as measured by the NPRS, at weeks 2 (mean difference: 1.26, *p* = 0.042, Cohen’s d = 0.69) and 5 (mean difference: 1.86, *p* = 0.004, Cohen’s d = 1.02) compared to the control group receiving standard care alone. Functional outcomes, assessed by the NFRS, were significantly better in the intervention group from week 1 through week 6 (*p* < 0.01), with large effect sizes (e.g., week 4: Cohen’s d = 1.22; week 8: Cohen’s d = 1.15). However, no significant differences were observed in WOMAC scores or state anxiety levels (STAI) between groups. These findings suggest that brief GI is a promising adjunctive intervention for enhancing pain management and early functional recovery in TKA patients.

### 4.2. Comparison with Prior Literature

The significant reductions in NPRS scores align with previous studies demonstrating the efficacy of GI in postoperative pain management. For instance, Jacobson et al. [12] reported improved gait velocity, reduced inflammatory markers, and lower WOMAC scores in TKA patients who used GI, though their intervention was longer. The moderate to large effect sizes observed in our study (e.g., NPRS at week 5: d = 1.02) are consistent with findings from a meta-analysis by Lee et al. [26], which highlighted the beneficial effects of mental training, including GI, on pain management post-TKA. Similarly, Choojaturo et al. [27] found that GI significantly reduced both pain and anxiety in TKA patients, though our study did not detect anxiety reductions, possibly due to differences in intervention duration or measurement timing.

The significant improvements in NFRS scores, with large effect sizes (e.g., d = 1.22 at week 4), underscore brief GI’s potential to enhance early functional recovery. This aligns with Kaplun et al.’s [10] findings in fibromyalgia patients, where brief GI improved pain, mood, and daily activities. However, the lack of significant WOMAC differences in our study contrasts with Jacobson et al. [12], potentially due to WOMAC’s lower sensitivity to short-term postoperative changes, as noted in prior TKA research [5]. The absence of STAI differences may reflect the low baseline anxiety levels in both groups, possibly attributable to the preoperative education program implemented at Barzilai Medical Center, as supported by Pinskiy et al. [15].

### 4.3. Clinical Implications

The findings suggest that brief GI, requiring only two minutes daily and delivered via smartphone audio recordings, is a feasible and effective intervention for TKA patients. The moderate to large effect sizes for pain (NPRS) and function (NFRS) indicate clinically meaningful improvements, particularly as the NPRS reductions at weeks 2 and 5 (1.26 and 1.86 points, respectively) approach or exceed the minimal clinically important difference (MCID) of 1.5–2 points for TKA patients [14]. These benefits could reduce reliance on analgesics, potentially lowering risks of opioid-related side effects, as supported by dos Santos Felix et al. [13]. The intervention’s simplicity and scalability make it suitable for integration into existing preoperative education programs, such as those described by Soever et al. [17] and Tilbury et al. [18], enhancing patient-centered care without significant resource demands.

The gender imbalance between groups (fewer men in the intervention group, *p* = 0.049) warrants consideration, as gender may influence pain perception or response to psychological interventions [10]. A post hoc ANCOVA adjusting for gender confirmed no significant interaction with pain or function outcomes (*p* > 0.05), suggesting that the observed effects of brief GI are robust. However, future studies should explore gender-specific responses to ensure equitable benefits.

In the context of contemporary pain science, the findings of this study align closely with the biopsychosocial model, which conceptualizes pain as an interaction of biological, psychological, and social factors. Guided imagery represents a psychologically oriented intervention that may influence pain perception by modulating attention, emotional responses, and cognitive appraisal of postoperative sensations. This is consistent with recent literature emphasizing the importance of integrating cognitive and emotional dimensions into musculoskeletal rehabilitation. Van Dijk et al. [28] highlighted that physiotherapists increasingly recognize the value of biopsychosocial approaches but often face barriers in implementing them systematically. Similarly, Samoborec et al. [29] demonstrated that psychological and social factors significantly contribute to non-recovery following musculoskeletal injury, underscoring the need for interventions that address more than tissue healing alone. The improvements in pain and function observed in our intervention group suggest that even a brief, low-intensity imagery protocol may positively influence postoperative recovery by targeting these broader mechanisms. This interpretation is further supported by Wijma et al. [30], who emphasized that addressing cognitive-emotional processes is a critical first step in effective pain neuroscience education. Thus, brief guided imagery may serve as a practical and scalable biopsychosocial tool that complements standard postoperative rehabilitation by engaging psychological pathways known to influence pain and functional outcomes.

### 4.4. Limitations

Several limitations must be acknowledged. First, adherence to the guided imagery protocol was not systematically measured using a daily log or automated monitoring tool. Although participants were instructed to practice daily and adherence was supported through weekly reminders, the exact frequency of practice is unknown. This limits our ability to quantify the dose of the intervention received. However, because randomization balances both measured and unmeasured factors between groups, the absence of precise adherence data does not compromise the internal validity of the randomized comparison. Second, this study was powered based on the primary outcome (NPRS), in accordance with standard clinical trial methodology; therefore, secondary outcomes such as WOMAC and STAI may be underpowered and should be interpreted as exploratory. Third, this study only assessed outcomes during the early postoperative period and therefore does not provide information about recovery beyond the 6-week follow-up. Fourth, the single-center design may limit generalizability to other clinical settings. Finally, the standardized multimodal analgesia protocol used in both groups [31] may have reduced the magnitude of between-group differences in pain, as both groups received effective pharmacological management.

### 4.5. Future Directions

Future research should evaluate this brief guided imagery protocol in larger and more diverse samples, ideally across multiple clinical centers, to strengthen external validity. Additional work is needed to determine the optimal delivery format, frequency, and duration of brief GI in the postoperative setting, as well as to identify patient subgroups who may benefit the most. Studies using controlled designs could also explore whether the short-term improvements observed here contribute to better recovery trajectories during the early and intermediate postoperative phases. Finally, integrating brief GI with other perioperative education or rehabilitation strategies may help clarify its role within a broader multimodal approach to postoperative care.

## 5. Conclusions

This study demonstrates that brief GI significantly reduces postoperative pain and enhances functional outcomes in TKA patients, with moderate to large effect sizes, though it does not impact anxiety levels. The intervention’s practicality and effectiveness support its integration into postoperative care to improve patient outcomes. While the intervention showed beneficial short-term effects, these findings should be interpreted with caution because adherence to daily GI practice was not systematically monitored. Further research is warranted to confirm these findings, optimize implementation strategies, and explore long-term benefits.

## Figures and Tables

**Table 1:** Demographic characteristics of the studied sample.

Variables	Control Group (N = 23)	Intervention Group (N = 19)	Comparisons
	Mean ± SD	Mean ± SD	
Age (years)	72.4 ± 7.2	69.9 ± 7.1	F = 1.25, *p* = 0.27
Years of pain	6.3 ± 4.3	6.7 ± 8.9	F = 0.038, *p* = 0.85
	N (%)	N (%)	
Sex (Men)	13 (56.5)	5 (26.3)	χ^2^ = 3.867 (df = 1), *p* = 0.049
Sex (Women)	10 (43.5)	14 (73.7)	χ^2^ = 3.867 (df = 1), *p* = 0.049

**Table 2:** Comparison between groups.

	NPRS	NFRS	STAI		WOMAC	
	Intervention	Control	Intervention	Control	Intervention	Control
T1	7.28 (2.34)	7.55 (2.34)	20.00 (13.52)	16.65 (13.69)	0.52 (0.20)	0.52 (0.24)
T2	7.66 (1.70)	6.98 (2.26)	24.27 (12.08)	25.35 (11.59)		
T3	7.03 (1.82)	7.52 (1.92)	15.80 (11.31)	18.30 (11.25)	0.39 (0.15)	0.48 (0.17)
T4	5.97 (2.27)	7.24 (1.33)				
T5	5.50 (2.31)	6.61 (1.62)				
T6	4.86 (1.95)	5.85 (1.79)				
T7	3.19 (1.59)	5.09 (1.92)				
T8	3.69 (2.07)	4.93 (2.29)				
	**Comparison**	**Comparison**	**Comparison**		**Comparison**	
Group	F (1, 36) = 3.25 η^2^_p_ = 0.08, *p* = 0.08	F (1, 39) = 10.83 η^2^_p_ = 0.22, *p* = 0.002	F (1, 33) = 0.001 η^2^_p_ = 0.00, *p* = 0.98		F (1, 37) = 0.76 η^2^_p_ = 0.02, *p* = 0.388	
Time	F (5.1, 183.65) = 27.22 η^2^_p_ = 0.43, *p* < 0.001	F (2.38, 92.84) = 21.91 η^2^_p_ = 0.36, *p* < 0.001	F (2, 66) = 5.79 η^2^_p_ = 0.15, *p* = 0.005		F (1, 37) = 5.45 η^2^_p_ = 0.13, *p* = 0.025	
Time ∗ Group	F (5.1, 183.65) = 2.36 η^2^_p_ = 0.06, *p* = 0.041	F (2.38, 92.84) = 6.29 η^2^_p_ = 0.14, *p* = 0.002	F (2, 66) = 0.78 η^2^_p_ = 0.02, *p* = 0.463		F (1, 37) = 1.57 η^2^_p_ = 0.04, *p* = 0.218	

Note 1. For each time point, the mean of the measurements is shown with the standard deviation in parentheses. Note 2. The *p*-values for the within-subjects factor and the interactions with the within-subjects factor of NPRS were calculated using the Greenhouse-Geisser correction to adjust for the violation of the sphericity assumption. Note 3. The *p*-values for the within-subjects factor and the interactions with the within-subjects factor of NFRS were calculated using the Huynh-Feldt correction to adjust for the violation of the sphericity assumption.

**Table 3:** Comparison between groups in pain measurements (NPRS) over time.

Time	Mean Difference Between Groups	Standard Error	Significance	95% Confidence Interval for Difference
1	0.26	0.77	0.733	(−1.29, 1.82)
2	−0.68	0.67	0.319	(−2.04, 0.68)
3	0.39	0.63	0.541	(−0.89, 1.67)
4	1.26 *	0.60	0.042	(0.05, 2.48)
5	0.86	0.61	0.168	(−0.38, 2.09)
6	1.23	0.61	0.051	(−0.01, 2.47)
7	1.86 *	0.60	0.004	(0.63, 3.08)
8	1.24	0.72	0.094	(−0.22, 2.71)

* Significant after Bonferroni correction (*p* < 0.05).

**Table 4:** Comparison between groups in function measurements (NFRS) over time.

Time	Mean Difference Between Groups	Standard Error	Significance	95% Confidence Interval for Difference
1	1.114	0.997	0.271	(−0.903, 3.131)
2	-	-	-	-
3	−1.607 *	0.681	0.023	(−2.986, −0.229)
4	−2.691 *	0.664	0.000	(−4.033, −1.348)
5	−2.050 *	0.664	0.004	(−3.392, −0.707)
6	−1.957 *	0.615	0.003	(−3.201, −0.713)
7	−2.452 *	0.681	0.001	(−3.828, −1.075)
8	−2.483 *	0.624	0.000	(−3.746, −1.220)

* Significant after Bonferroni correction (*p* < 0.05).

## Data Availability

The raw data supporting the conclusions of this article will be made available by the authors on request.
